# Chemical Contaminants in Cerumen Samples from Ecuadorian Stingless Bees: Reporting Glyphosate, Aminomethylphosphonic Acid, and the Presence of Metals and Metalloids

**DOI:** 10.3390/insects16111079

**Published:** 2025-10-22

**Authors:** Joseline Sofía Ocaña-Cabrera, Jorge Ron-Román, Sarah Martin-Solano, Claude Saegerman

**Affiliations:** 1 Research Unit of Epidemiology and Risk Analysis Applied to Veterinary Sciences (UREAR-ULiège), Fundamental and Applied Research for Animal and Health (FARAH) Centre, Faculty of Veterinary Medicine, University of Liège, Quartier Vallée 2, Avenue de Cureghem 6, B43a, 4000 Liège, Belgium; jocana@doct.uliege.be; 2 Grupo de Investigación en Sanidad Animal y Humana (GISAH), Carrera de Ingeniería Agropecuaria, Departamento de Ciencias de la Vida y de la Agricultura, Universidad de las Fuerzas Armadas ESPE, Campus Politécnico Hacienda el Prado Selva Alegre, Sangolquí 171103, Ecuador; jwron@espe.edu.ec; 3 Grupo de Investigación en Sanidad Animal y Humana (GISAH), Carrera de Ingeniería en Biotecnología, Departamento de Ciencias de la Vida y de la Agricultura, Universidad de las Fuerzas Armadas ESPE, Av. Gral. Rumiñahui S/N, Sangolquí 171103, Ecuador; ssmartin@espe.edu.ec

**Keywords:** cerumen, Meliponini, environmental pollution, health risks, hazard quotient

## Abstract

**Simple Summary:**

Stingless bee cerumen is made of wax and plant resins. While collecting materials, these Meliponine bees may pick up chemical contaminants, which can enter their nests. This study investigated chemical pollutants in Ecuadorian cerumen, focusing on glyphosate (GLY), aminomethylphosphonic acid (AMPA), pesticides, metals, and metalloids. Researchers used advanced chromatography techniques to detect contaminants. Glyphosate and AMPA were found in samples from the highlands, while metals and metalloids were detected in both the Amazon and highland regions. No other pesticides were found. The risks to humans are minimal, though one stingless bee species (*Tetragonisca angustula*) may be more affected. Cerumen could be useful for monitoring environmental pollution. Clear guidelines are needed for its safe use and production.

**Abstract:**

Stingless bee cerumen is a mixture of wax and plant resins. Foragers of stingless bees are exposed to various chemical contaminants during their plant visits and collection activities. These contaminants have the potential to be transferred into the nest. This study aimed to elucidate the existence of chemical contaminants in Ecuadorian cerumen. To this end, the following aims were established: (i) to determine and quantify glyphosate (GLY), aminomethylphosphonic acid (AMPA), some other pesticides, metals and metalloids in cerumen and (ii) to establish possible risks associated with the presence of these chemical contaminants to the health of stingless bees and humans. The quantification of chemical contaminants was conducted using gas chromatography (GC), liquid chromatography (LC), and ion chromatography (IC) coupled to mass spectrometry (MS). Glyphosate (0.02–0.2 mg/kg) and AMPA (0.028 mg/kg) were detected in four of the pooled samples (n = 14) from the northern and southern highland regions. Other pesticide traces were not detected in any cerumen samples. Metals (Cd, Cr, Pb, Ni, Sn) and metalloids (As, Sb, Se) were found in all samples, including highlands and the lower Amazon. The potential risks of exposure to glyphosate and AMPA for stingless bees and humans appear to be minimal (except for the specific conditions given for *Tetragonisca angustula*) and safe, respectively. It seems that cerumen may serve as an effective biomonitoring matrix for assessing the environmental health of stingless bee nests. Establishing guidelines and regulations for the safe use and handling of products derived from the stingless bee consumption is therefore imperative.

## 1. Introduction

The term “cerumen” is used to describe a malleable structural material that is used by stingless bees to build, repair, adapt [1], and protect [2] their nests. The cerumen is composed of wax and plant resins [3]. According to the stingless bee species, cerumen is part of the involucrum, pillars, storage pots [4], brood cells [5], and the imprisonment chamber [6], which are located at the inner level of the nest. In addition, in certain stingless bee species, the cerumen is part of the entrance refinements at the outer level of the nest.

From a phytochemical perspective, resins are plant-derived secondary metabolites—comprising terpenoids, phenolics, and fatty substances [7]—that either form on plant surfaces or internally; in stingless bee colonies, these resins play a crucial protective role by deterring predators and intruders [8], while also contributing to the stingless bees’ cuticular chemical profiles [9] aiding in colony member recognition. The proportion of the workforce engaged in resin collection has been estimated to be less than 10% of the total workforce in the *Trigona* genus [10], and up to 50–90% in other genera [11,12]. In the Neotropics, these figures are less than 20% of the total workforce in the *Melipona* genus [13,14]. The intensity of resin collection is influenced by several factors, including specific stingless bee species, weather conditions, seasonal variations, internal colony needs, external resource availability [15,16,17].

The foraging activity of stingless bees can transfer contaminants from the surrounding environment to the nest and subsequently to pot-honey and other stingless bee byproducts. In areas where foraging grounds for stingless bees were polluted, several undesirable chemicals may enter stingless bee products via the nectar, pollen, or sugary exudates of plants that were grown there [18]. Plants can accumulate chemical pollutants in their tissues through various processes, including soil and water absorption and atmospheric adsorption. The bioaccumulation of these chemical pollutants is a gradual process, and as a result, they become biomagnified within the food chain [19].

The indiscriminate use of agrochemicals represents a growing threat to the sensory and cognitive abilities of foraging honey and wild bees [20,21,22,23]. Exposure to sub-lethal doses of pesticides has been demonstrated to compromise the neuronal plasticity of stingless bees during ontogenesis [24]. This has been shown to reduce the brain volume of worker stingless bees, particularly in the mushroom bodies and optic lobes, which has a detrimental effect on their foraging performance [25].

Due to the exposure to multiple agrochemicals through various routes in the field, stingless bees may be more susceptible to toxic effects, suggesting a need for more comprehensive toxicological experiments. A study conducted in Brazil evaluated the lethal and sublethal toxicity of various agrochemicals on an endangered native Brazilian bee species, *Melipona* (Michmelia) *capixaba* Moure & Camargo, 1994 [24]. Thiamethoxam induced a high mortality rate in this species of stingless bee, irrespective of the exposure route or dosage. Furthermore, a shift in the flight capacity was detected in response to the lowest observed dose via contact exposure. The administration of a sub-lethal dose of glyphosate resulted in elevated mortality rates following oral exposure, in addition to a significant impairment in the flight capacity of *M. capixaba* following contact exposure [22].

Despite the superiority of research conducted on honey bees compared to stingless bees regarding pesticide toxicity, most studies on the lethal effects of pesticides on stingless bees have focused on adults. However, experimental evidence suggests that ingestion of pollen and nectar contaminated with neonicotinoids and organophosphates can affect the health of larval stingless bees. The effects of exposure of the larvae of *Scaptotrigona bipunctata* to different doses of chlorpyrifos (an organophosphate compound) resulted in the production of lighter, smaller, and deformed adult workers [26]. The exposure to neonicotinoid products during the larval stage results in alterations to the brain [27]. On the other hand, the risks associated with exposure and contact with chemical pollutants, such as parathion, benomyl, and arsenic pesticides, can affect human health [28], and others, such as DDT, dieldrin, heptachlor [29,30], and neonicotinoids, can affect animal and environmental health [31,32].

Chemical compounds, including pesticides, herbicides, volatile organic compounds (VOCs), metals, and metalloids, can be defined as chemical pollutants [33]. However, metals and metalloids differ from other contaminants in terms of their environmental impact. Metals and metalloids are continuously emitted from both natural sources and anthropogenic activities. As they do not degrade, they enter into physical and biological cycles [34]. On the other hand, pesticides are dispersed in time and space by human action and, depending on the main chemical compound, are degraded by various environmental factors over a longer or shorter period [35].

Pesticides can become persistent environmental pollutants [36]. Among the most widely studied of these is glyphosate (GLY), due to its widespread use and the perceived threats to the ecosystem, human [37], and animal health [38]. Glyphosate, N-(phosphonomethyl) glycine, a broad-spectrum herbicide, is primarily used for weed control and canopy clearing before seeding [39]. Glyphosate can be degraded via bond cleavage. The C-N bond cleavage forms aminomethylphosphonic acid (AMPA), a persistent metabolite in the environment [40]. The C-P bond cleavage of glyphosate forms sarcosine and glycine, safer metabolites [41]. The photodegradation of amino-polyphosphonates [42] and the textile and paper industries [42] are other sources of AMPA. Aminomethylphosphonic acid accumulation has been reported in a variety of species, including plants [43], chickens [44], fish [45], and honey bees’ wax [46].

Fungicides, pesticides, and herbicides contain a range of metals and metalloids, including iron (Fe), manganese (Mn), and arsenic (As). The recurrent utilization of these chemical compounds fortified with metals and metalloids, fertilizers, and organic matter, including sludge and sewage, can potentially result in extensive contamination, exemplified by elevated copper (Cu) and zinc (Zn) concentrations in soils employed for the cultivation of citrus and other fruit crops [47]. Some trace metals, such as cadmium (Cd) and lead (Pb), enter the soil as fertilizer impurities [48]. Several anthropogenic activities contribute to the release of nickel (Ni) and chromium (Cr) into the environment, including iron and steel production, food processing, waste incineration, fertilizers, mining and metallurgy, the textile industry, paints and pigments, tanning, the chemical industry, and the leather industry [49,50]. Metals such as Pb [51], Zn, Cr, Cd, and metalloids such as As [52] have been reported in pot-honey, propolis [53], geopropolis [54], pot-pollen, beebread, and wax [55] from stingless bees. Studies of metals, metalloids, and pesticides in cerumen (a mixture of wax and plant resins) in stingless bees are, to the best of the authors’ knowledge, scarce or non-existent. The present study included the detection of seven metals: cadmium (Cd), chromium (Cr), copper (Cu), mercury (Hg), tin (Sn), nickel (Ni), and lead (Pb), and three metalloids: antimony (Sb), arsenic (As), and selenium (Se).

The accumulation of pollutants in bee products can be attributed to the prevalence and persistence of these chemical compounds in the environment [56]. Additionally, bees collect chemical contaminants from plants (nectar, pollen, and resins) that have been contaminated with pesticides, metals, metalloids, and persistent organic pollutants (POPs) [57]. Meliponiculture, an economic activity that consists mainly of collecting products derived from stingless bees for human use, is a topic that continues to gain recognition worldwide. However, there is currently no regulation in place, for example, within the Codex Alimentarius, regarding the quality or safety of these types of products. Brazil has developed national regulations, but these only apply to pot-honey from the *Melipona* genus. Ecuador has no such regulations. For this reason, Brazilian regulations will be used for comparison wherever possible, alongside references to European honey bee products and World Health Organization (WHO) regulations on the risk of human contaminant intake.

The present study was thus designed to elucidate the existence of chemical contaminants in stingless bee nest products. The specific objectives were as follows: (a) to determine the presence of glyphosate, its metabolite aminomethylphosphonic acid (AMPA), other pesticides, metals, and metalloids in cerumen; and (b) to establish possible risks associated with the presence of these chemical contaminants to the health of stingless bees and humans.

## 2. Materials and Methods

### 2.1. Sample Geographical Description

**Figure 1:**
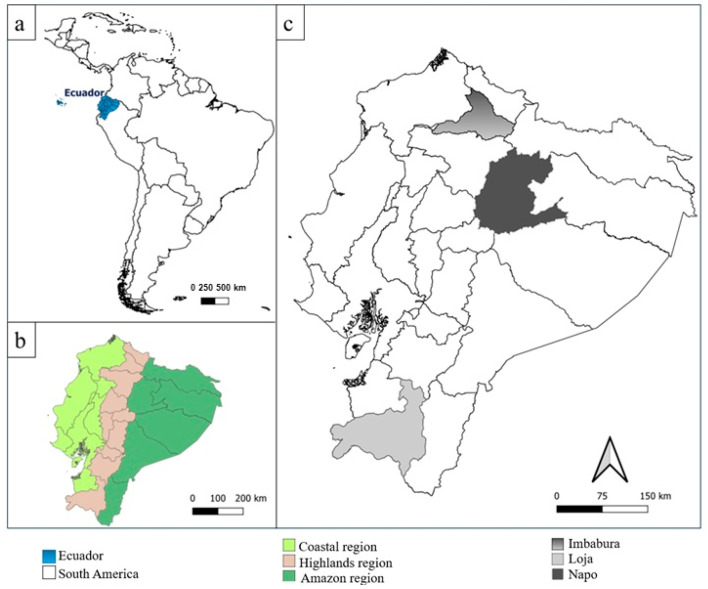
(**a**) Geographical location of Ecuador in South America. (**b**) Natural regions of continental Ecuador. (**c**) Sampled provinces of Ecuador.

Cerumen samples were obtained from ten localities in two distinct geographical regions: the Amazon and the highlands. Cerumen samples (n = 41) were collected in March 2019 in two highlands provinces, Loja province (seven localities), in August, September, October, and December 2023, and in July 2023 in Imbabura province (one locality) (Figure 1). Cerumen samples were also taken in January 2024 from Napo province (two localities) in the Amazon region. Cerumen samples were taken from storage pots and involucrum during the transfer of natural nests to wooden boxes for easier handling, and from nests in wooden boxes that had been established for a long time. The amount taken varied according to each nest, and care was always taken not to deprive the colony of this important material.

To fulfil the requisite minimum weight for laboratory analysis of cerumen and reduce costs, a method of pooling the cerumen was employed. The composition of the pools was primarily determined by the proximity of the samples (Table 1).

### 2.2. Chemical Contaminants Selected for Analysis

A total of seventeen pesticides (Table 2) were selected for detection and quantification in cerumen samples, including GLY and AMPA, as well as seven trace metals: Cd, Cr, Cu, Hg, Sn, Ni, and Pb, and three trace metalloids: Sb, As, and Se. Note that Se is one non-metal but sometimes considered as a metalloid. We encapsulated Se as a metalloid in this paper. The rationale behind this selection was twofold: firstly, the recurrent use and reporting of these pesticides in the country, and secondly, the importance of stingless bees as pollinators in the region [58,59,60].

### 2.3. Chemical Extraction and Detection Process

The analysis was carried out in July 2024 by the GIRPA laboratory (France), COFRAC ESSAIS accredited under the number N° 1-6951 for laboratory analysis by the French competent authority.

#### 2.3.1. Sample Preparation for Multi-Residue Analysis

Cerumen samples (0.25 g) were homogenized using ceramic homogenizers and transferred into a 7 mL polypropylene (PP) tube. A 5 mL solution of isopropanol/hexane (50/50, *v*/*v*) was added and shaken for one minute. After freezing for an hour, the tube was centrifuged (9000× *g*, 5 °C), and the supernatant was evaporated to form an oily residue. The extract was reconstituted with acetonitrile, then dispersed, and MgSO_4_ + PSA was added and shaken. After centrifugation, an aliquot of the supernatant was diluted with water and filtered before LC-MS/MS analysis (LC Exion system and API 7500 triple quadrupole mass detector (Sciex, Framingham, MA, USA), column C18 Synergi Hydro-RP 100 mm × 3 mm; 2.5 µm (Phenomenex, Torrance, CA, USA)). An additional aliquot of the supernatant was concentrated (nitrogen stream) and reconstituted with ethyl acetate before GC-MS/MS analysis (Chromatograph 8890 and 7010 quadrupole mass detectors (Agilent Technologies, Santa Clara, CA, USA), columns 1 and 2 HP-5 MS (15 m × 0.25 mm ID; 0.25 µm) (Agilent Technologies)).

The limit of quantification (LOQ) was 20 µg/kg for cypermethrin, flonicamid, malaoxon, thiacloprid, 50 µg/kg for diafenthiuron, imidacloprid, pyrethrin I, and 10 µg/kg for the other ten pesticides.

The validation method was based on five recoveries at LOQ plus five recoveries at 10×LOQ. Appendix B and Appendix C show LC-MS/MS (acetamiprid, benomyl, carbendazim, methomyl, flonicamid, thiacloprid, thiamethoxam, malaoxon, malathion, chlorantraniliprole, trichlorfon, pyrethrin I, imidacloprid, diafenthiuron), GC-MS/MS (diazinon, cypermethrine, deltamethrine), detection parameters, and detailed validation data.

#### 2.3.2. Sample Preparation for Glyphosate (GLY) and AMPA Detection

A 0.25 g sample was weighed in a 7 mL PP tube with ceramic homogenizers. A radiolabelled glyphosate 13C-15N standard was added. 1.25 mL of methanol/formic acid (100/1, *v*/*v*) was added and shaken for 30 s. Another 1.25 mL of ultrapure water was added, and the tube was agitated for 30 s. The tube was centrifuged (3000× *g*), and 2 mL of the supernatant was collected and washed with 1 mL of hexane. The hexane layer was discarded, and an aliquot of the extract was filtered before LC-MS/MS analysis.

For both compounds, the limit of quantification (LOQ) was 10 µg/kg. The validation method was based on five recoveries at LOQ plus five recoveries at 10×LOQ. For LC-MS/MS (LC Exion system (Sciex) and API 6500+ triple quadrupole mass detector (Sciex), column Metrosep A, Supp 5 (150 mm × 4 mm ID; 5 µm) (Metrohm, Herisau, Switzerland)), detection parameters, and detailed validation data, see Appendix D.

#### 2.3.3. Sample Preparation for Metals and Metalloid Trace Detection

A microwave-assisted acid digestion process was conducted on 0.25 g of the homogenized sample with 10 mL of concentrated ultrapure nitric acid (ω ≥ 67%). A microwave-assisted heating program was applied, utilizing a ramp time of 25 min and a holding time of 10 min at 240 °C. Upon cooling, the extract was diluted to 50 mL with ultrapure water. IC-MS was then used to trace inorganic analytes. We detail the LOQ limits along with the concentrations found in the Section 3.

The validation method was based on five recoveries at LOQ plus five recoveries at 10×LOQ. See Appendix E for details about the limit of quantification, IC-MS (Model 7850 (Agilent Technologies), automatic sample changer model SPS 4 (Agilent Technologies), and micromist Nebulizer (Agilent Technologies)), detection parameters, and detailed validation data.

### 2.4. Statistical Analysis

The Dunn–Kruskal–Wallis multiple comparison test and *p*-values adjusted with the Bonferroni correction were employed to identify significant differences between the mean ranks of each chemical contaminant concentration according to the sampling location (Ecuadorian region). The univariate analysis (General Linear Model) was used to ascertain a correlation between the detection of chemical contaminants and the geographical locations from which they were sampled.

### 2.5. Risk Assessment of Glyphosate Exposure in Stingless Bees

The cerumen “pools” sampled in this study indicate the environmental health surrounding the meliponaries (nest set) in the localities under investigation. It is interesting to provide a preliminary estimation of the possible impact of glyphosate, as detected in cerumen samples in this study.

To estimate the risk for stingless bees of exposure to glyphosate we used the Risk Quotient (RQ) (Equation (1)) as the ratio between an exposure estimate, in this study defined as the concentration of the residue (mg/kg) and a point estimate of effect, in this study defined as the acute oral LD_50_ (expressed as µg/bee or µg/L) Table 3.

Risk Quotient (RQ) = Exposure/Toxicity = (Estimated concentration of GLY in cerumen)/(LD_50_ of GLY for stingless bees), (1)

For this study’s screening level assessment of GLY, the Limit of Concern (LCO) was defined as follows: LCO = 0.4 for acute exposure and LCO = 1 for chronic exposure [62]. If the obtained RQ values were below their respective level of concern, it can be presumed that the risks to stingless bees were minimal.

We set 4 g of cerumen as the amount handled/chewed by stingless bees [63]. The body weight of stingless bees was set at 4.68 mg for *Tetragonisca angustula* [64].

### 2.6. Glyphosate Human Exposure Risk Assessment

According to the reports of Paredes (2022) [65], based on 14 personal interviews and the personal communication conducted by the authors of this study with 16 Ecuadorian stingless bee keepers and their families, cerumen has the following main uses: cleansing rituals involving burning of cerumen within the domestic environment, eating honey-pots [66,67], the application of cerumen slides on the scalp of neonates, and direct application to the skin in regions where an injury has occurred. The potential for adverse human health effects can arise through exposure by inhalation, ingestion, and dermal contact [68].

To estimate the human health risk of exposure (Equation (2)) to GLY, we used two toxicological safety thresholds established by EFSA in 2015 and currently applicable [69], and adjusted to the reality of meliponiculture in Ecuador. In this study for healers [65], we set 1.5 mg/kg human body weight per day for the acute reference dose (ARfD), and for consumers [70,71], we set 0.5 mg/kg human body weight per day for the acceptable daily intake (ADI).

To estimate the amount of cerumen daily handled/chewed, we used 0.80 g for the weight of a cerumen pot as the minimum unit of handled/chewed (the pot weight is closely related to the stingless bee species) [72,73]. The average weight of the Ecuadorian population that may be exposed to these risks is exemplified as follows: 67.9 kg for men, 62 kg for women [74], and 3.2 kg for newborns [75].

We have posited a worst-case scenario to assess the risks of the daily consumption/skin contact of cerumen pots for human health. We multiplied the highest levels of consumption/skin contact of cerumen by the highest GLY and AMPA residues quantified in pooled samples and added a factor for the dilution required to prepare the pools (i.e., for pool 1, the factor was 10).

(2) $$\mathrm{Human}\;\mathrm{health}\;\mathrm{risk}\;\mathrm{assessment}=(\mathrm{GLY}\;\&\;{\mathrm{AMPA}}_{\mathrm{concentration}}\frac{mg}{{kg}_{cerumen}})\;\times \;\;\mathrm{consumption}/\mathrm{skin}\;\mathrm{contact}\;\mathrm{rate}\;\frac{{kg}_{cerumen}}{{kg}_{body\;weight}\;per\;day}\;\times \;\mathrm{dilution}\;\mathrm{factor}$$

Ultimately, the levels of glyphosate exposure were compared with the toxicological reference values previously mentioned to describe the potential risk.

## 3. Results

Fourteen “pools” of cerumen samples were analyzed. Samples were obtained from two regions, 3 provinces, 10 localities, 17 meliponaries, and 41 stingless bee nests. A comprehensive account of the residues of contaminants quantified in cerumen pools is presented in Table 4.

The results of the multi-residue analysis did not identify any of the 16 agrochemicals analyzed.

### 3.1. Metal and/Metalloids Trace Detection

The minimum number of chemical compounds (metals, metalloids, glyphosate, and AMPA) detected in each sample was 3/10, with a maximum of 9/10. The number of compounds identified in each region was as follows: southern highlands 6/10, northern highlands 9/10, and low Amazon 5/10.

Each compound’s mean concentration ± standard error (minimum and maximum values) was calculated and presented in mg/kg. The main results for arsenic (As) were 0.133 ± 0.087 (0.028–0.27), chromium (Cr) 1.528 ± 2.304 (0.11–7.1), and lead (Pb) 0.247 ± 0.393 (0.031–1.5).

A single pool was identified as having tin (Sn) and selenium (Se). The positive pools were five and three, with respective concentrations of 0.17 mg/kg and 0.16 mg/kg (see Table 4).

### 3.2. Glyphosate (GLY) and AMPA Detection

The mean concentration of glyphosate ± standard error (minimum and maximum values) was 0.096 ± 0.094 mg/kg (0.014–0.2) in pools 1, 2, 3, and 5 from northern and southern highlands, while the mean concentration of AMPA was 0.028 mg/kg in pool number five (northern highlands). It was noted that this pool contains both GLY and AMPA.

Significant differences were observed in the mean rank concentrations of arsenic, chromium, and lead (Dunn Test with Bonferroni correction, *p*-value = 0.04, 0.03, and 0.02, respectively) between the low Amazon (Napo) and northern highlands (Imbabura) regions. The detection of glyphosate in cerumen samples from north and southern highlands did not yield statistically significant differences (Table 5). The latter compound was not detected in the low Amazon region.

### 3.3. Risk Assessment of the Presence of Agrochemicals and on the Health of Stingless Bees and Humans

Metals such as cadmium and metalloids such as antimony and arsenic appear to be associated with the geographical region of Ecuadorian habitats of stingless bees (Table 6).

In our study, the stingless bee genera represented in the glyphosate and AMPA-positive cerumen (pools 1, 2, 3, and 5) were the genera *Melipona*, *Scaptotrigona*, *Partamona*, and *Trigona*. To illustrate the RQ calculation, we developed a *T. angustula* worst-case scenario. The last means a cumulative exposure of GLY (the highest concentration of this study) and AMPA (the only concentration identified in this study) over *T. angustula* foragers. The toxicity was set at 0.015 µg a.i./bee (see Table 3 and the methodology for handling and chewing). Exposure calculation included (0.02 + 0.028) mg GLY + AMPA/kg cerumen * 0.004 kg cerumen = 0.000912 mg GLY+AMPA. The toxicity calculation included 0.000015 mg a.i. for GLY/*T. angustula*. The estimation of RQ was equal to 60.8. Considering the 95% CI for the 48 h oral LD_50_, the RQ is between 22.8 and 182.4. This RQ represents a case of acute exposure [60].

Using the highest concentration of glyphosate found in this study, 0.2 mg/kg, and the highest cerumen handled and chewed, based on 0.80 g of cerumen and the average weight of Ecuadorian neonates, women, and men, none of the three toxicological safety thresholds exceeded the ADI and ARfD thresholds established by EFSA [70].

## 4. Discussion

Stingless bee cerumen is sometimes compared to honey bee propolis, but cerumen serves as the primary building material in the nest and is continuously reworked and recycled. A more accurate comparison is with honey bee wax, part of a permanent honeycomb structure [81]. In addition, in some Latin American communities, cerumen, as a by-product of stingless bees, has been used in folk medicine to improve human health, typically in the form of infusions added to beverages [71].

### 4.1. Glyphosate (GLY) and AMPA in Stingless Bees’ Cerumen

Glyphosate is a globally used herbicide [39], and as a pesticide, it is a pollutant that harms non-target organisms. There is no consensus on its toxicity among specialists around the world. Glyphosate is absorbed across the leaves and stems and is translocated throughout the plant [82]. The foraging activities of stingless bees may result in the inadvertent introduction of agrochemicals applied to plants into nests [83].

AMPA, the major degradation metabolite of glyphosate, along with glyphosate itself, persists in the environment due to its binding to soil particles; thus, they are accumulated in surface layers and dispersed via various means, including surface runoff, windborne dust, and vertical transport in the soil to groundwater and aquatic sediments [84,85]. There is evidence to suggest that the presence of glyphosate in soil can enhance microbial activity [86], including bacteria involved in its degradation process (*Bacillus* and *Proteus*) [87]. These bacteria are phylogenetically related to bacteria found inside the nests of four stingless bee species in Brazil, which are associated with larval development [88]. It can thus be postulated that the presence of these bacteria in the stingless bees’ nest, in conjunction with specific environmental conditions, contributed to the degradation of glyphosate to AMPA in cerumen as a matrix. However, further research is required to ascertain the potential role of cerumen bacteria in the degradation of this herbicide.

Glyphosate and AMPA residues were found in beeswax samples, reporting that those maximum concentrations may cause sublethal effects in honey bees [46]. Wax is primarily composed of hydrocarbons and can act as a lipid sink for lipophilic pesticides. In contrast, glyphosate and AMPA are polar pesticides. This herbicide gains access to a matrix such as cerumen in two ways: indirectly, via stingless bees’ foraging activities, and directly, due to its solubility in compatible compounds, including terpenoids and hydrophilic phenols, which may be present in plant resins.

The toxicity of a pesticide may vary depending on the bee species [89,90]. However, in the case of *Partamona helleri*, a stingless bee species belonging to one of the genera represented in this study, acute oral exposure to commercial formulations of glyphosate resulted in increased glutathione S-transferase (GST) activity and 100% survival after exposure to 7400 µg/mL of herbicide. Although its potential to alter feeding behaviour, resulting in a decrease in food intake, is known [91]. Two of the samples in this study, in which glyphosate was detected, 0.15–0.2 mg/kg, exceeded the permitted limit for European honey from honey bees, 0.05 mg/kg.

Adult bees of the *Tetragonisca angustula* exposed to concentrations of 400 µg/L, which were found in water bodies close to soybean crops, showed no short-term mortality effect. However, when the stingless bee *T. angustula* directly consumed 178 g/L of the product, they died rapidly [92]. A recent study estimated a 48 h oral LD_50_ for *T. angustula* foragers to equal 0.015 µg a.i./stingless bee, with a 95% CI: 0.005–0.040 [61]. This has been observed to affect the worker’s locomotion, behaviour, and biology. Further research is required on the various stingless bee species to elucidate their potential impact on survival. Furthermore, additional research is essential to examine lethal and sublethal glyphosate effects in conjunction with other pesticides. For example, studies should investigate the impact of glyphosate on larval mortality in *Melipona scutellaris* [93]. This may provide a more comprehensive explanation of the acute risk quotient (RQ) values found in this study and why they exceed the Level of Concern according to the Guidance for Assessing Pesticide Risks to Bees [62].

Although the estimated thresholds of human health risks from glyphosate exposure in this study did not appear to be cause for concern, further research is recommended. Humans can be exposed to glyphosate and AMPA particles daily. This exposure can occur through the inhalation of dust particles present in domestic environments. The concentration of these particles is influenced by several factors, including the geographical location of the residence (rural or urban), the proximity of crops, and the use of herbicides on driveways or lawns [94]. Glyphosate and its metabolites have been linked to a slight increase in the incidence of non-Hodgkin lymphoma, as well as to the induction of genetic damage, increased oxidative stress, interference with the estrogenic pathway, and altered brain function. It also has mutagenic/carcinogenic potential and endocrine-disrupting effects (EDEs) on the human reproductive system [68].

### 4.2. Metals, Metalloids, and Micronutrient Elements in Cerumen

Arsenic (As) concentrations are strictly related to geographical locations. The most toxic forms of arsenic are inorganic arsenic (As III) and arsenic (As V). The latter is the form of arsenic associated with bioaccumulation in plant pollen or nectar, which subsequently enters stingless bee nests [95]. The concentration of arsenic in European honey bee propolis was found to be in the range of 0.075 to 0.66 µg/g [96,97], a reliable arsenic indicator, especially compared to honey from honey bees. The concentration of arsenic found in this study falls within the range above, suggesting the usefulness of stingless bee cerumen as a good indicator of metalloids in the environment. *Apis mellifera* honey samples from Chile showed total arsenic concentrations between 0.0022 and 0.1719 mg/kg, and up to 0.0246 mg/kg of inorganic arsenic, with honey being a good indicator of environmental pollution [98]. Volcanoes and their emissions, mineralised zones, fluid migration, gas emissions (particularly in regions of geographic faulting), and hydro(bio)geochemical processes are common sources and routes of arsenic in Latin America. These sources and routes are directly responsible for the discharge of arsenic into near-surface ecosystems, where it contaminates soil, water, dust, and aerosols, as well as living organisms, plants, and humans [99]. Forager bees exposed to the highest concentrations of arsenic have severe growth defects and deficits in learning and memory [100]. The human health effects of chronic arsenic poisoning include cardiovascular disease and neuropathy. In women, they include miscarriage, premature birth, and low birth weight, even at low levels of exposure [101].

A high concentration of cadmium (Cd) in soil results in increased plant uptake, leading to elevated human exposure through the consumption of fruits and vegetables [102]. Cocoa plantations in coastal and Amazonian regions of Ecuador clearly illustrate it [103], which are endemic to areas with stingless bee populations. In these areas, cocoa beans were labelled to exceed European Cd guidelines. The findings of Barraza et al. (2017) [104] indicate that agricultural practices, rather than oil activities, are the primary source of Cd in Ecuadorian soils. This could explain the higher concentrations of Cd observed in cerumen, 0.64, 1.4, 4.4 mg/kg, from areas of intensive agriculture in the country’s southern highlands, which also exceed the limit, 0.10 mg/kg, set by Brazilian legislation for stingless bee honey.

Lead (Pb) is a naturally occurring metal. Humans are exposed to it through inhalation, ingestion, and dermal contact [105]. In the Amazonian region of Ecuador, lead human contamination through food and water is associated with oil exploitation [106] and mining in southern provinces. The lead concentrations found in the cerumen of the stingless bee genera *Scaptotrigona* and *Partamona* from the Ecuadorian highlands exceeded the European safety guideline of 0.10 mg/kg for honey produced by honey bees [79] and also those set by Brazilian legislation for stingless bee honey [76,77]. In Brazil, samples of “samburá” (fermented pollen) and wax from *Melipona subnitida* had high Pb concentrations (7.4–1.3 mg/kg, respectively) [55].

The U.S. Food and Drug Administration (FDA) prioritizes the assessment of cadmium (Cd) and lead (Pb) to ensure food safety, given the potential harm that these metals can cause to human health, particularly during human brain development [107]. According to the latter guidelines, the Pb and Cd concentrations in the cerumen samples from the highlands of this study also exceed the established limits for *A. mellifera* honey (0.30 and 0.10 mg/kg, respectively). After 10 days of exposure to Cd and Pb, a constant bioaccumulation was observed in the bodies of honey bees, along with an association with higher levels of α-tocopherol (the most active form of vitamin E, with antioxidant function) [108].

The levels of cadmium found in different honey bee and stingless bee products in different Latin American countries are as follows: 0.070–2.875 mg/kg in Peru [109]; 2.1–3.4 mg/kg in Mexico [110]; 0.008–0.009 mg/kg in the Amazon region and southern Brazil [111]; and 0.044 mg/kg in Chile [112]. Our results are comparable with those for the lowest concentrations, such as in the Amazon, and the highest concentrations, such as in the Highlands. This confirms that the detection of this metal depends heavily on the region and the anthropogenic activities that may be the source of these metals in each area. Lead concentrations in bee products in Peru: 0.001–2.478 mg/kg [108], 0.015–0.148 mg/kg in Brazil, and 0.339 mg/kg in Chile [111] show that concentrations of this heavy metal in the samples studied remain relatively similar to those reported.

Chromium (Cr III) is essential for all living organisms, including humans [113]. The contamination of land and waterways with hexavalent chromium has increased as a consequence of human activity, including the processing of metals and metalloids and leather, the textile industry, steel welding, and the use of pigments [114]. Furthermore, it is a relatively soluble anion that is highly active and mobile in soil matrices [115]. The contamination of the environment with chromium is a significant threat, particularly to water and soil [116], which are two of the main resources for stingless bee nests. Our analysis revealed that nine of fourteen cerumen samples exceeded the Cr limits set forth by Brazilian legislation of maximum limits for inorganic contaminants in foodstuffs, which also applies to pot-honey from stingless bees [18]. The chromium concentration in honey bees in Mexico was found to vary between 0.39 and 1.84 mg/kg [109] while the concentration in bee products from the same country varied between 0.001 and 4.52 mg/kg [117]. Concentrations in honey from stingless bees in Brazil range from 0.28 to 0.51 mg/kg [18]. The reported concentrations are similar to, and vary as much as, the concentrations observed in our study, depending on the geographical area.

The levels of chromium (Cr) that are acutely lethal are significantly higher than those normally found in bee matrices and the environment. This suggests a moderate risk of chromium in real-world scenarios for wild pollinators. However, chronic effects include impacts on larval development and cognitive impairment, similar to those observed with Pb [118]. Chromium (III) is an essential nutrient for humans as it plays a role in carbohydrate and fat metabolism. In contrast, Cr (VI) can lead to respiratory irritation, lung cancer, skin ulcers, and kidney and liver damage. Exposure to Cr (VI) can damage DNA and cellular repair mechanisms [119].

In Ecuador, contamination of crops by non-essential metals and metalloids has been a subject of growing concern over the past three decades, particularly in the Andean highland region, where several potentially contaminating elements from volcanic and subterranean sources, such as Tin (Sn) and Selenium (Se), were mentioned [120]. Nickel (Ni) and Antimony (Sb) are both present in the environment and dispersed due to anthropogenic activities, for example, in sulphur compound mine drainage waters [121], and in human wastewater due to their use in treatments against human parasites [122], and the discharge of metals and metalloids in the form of gunshot residues (barium, antimony, and lead) due to military activities [123] or to hunting.

The foraging behaviour of stingless bees is responsive to environmental changes, particularly those related to pollutants such as metals and metalloids. Stingless bees serve as reliable indicators of environmental quality, a method known as biomonitoring [18]. However, the absorption of metals and metalloids alters the magnetic particles in stingless bees, affecting their orientation and food-foraging behaviour [124,125].

For humans, the primary routes of exposure to particles of substances such as arsenic, cadmium, lead, thallium, and mercury are ingestion, skin contact, or inhalation [126,127]. Excessive exposure to metals and metalloids in human internal tissues (absorption) can impact the central nervous system and act as a pseudo-cofactor or promoter of several illnesses [33].

The study areas in the northern and southern highlands of the country showed the highest levels of glyphosate/AMPA, metals, and metalloids, which is evidence of the unhealthy environment for stingless bees in these areas, where agriculture and mining are the main sources of pollution. Detecting glyphosate in cerumen samples inside stingless bee nests may represent a risk to nest health.

### 4.3. Absence of Several Other Pesticides in Stingless Bee Cerumen

Toxic contaminants in both managed and wild colonies of stingless bees prove that agrochemicals are a significant factor in their decline. Soil-applied chemicals are absorbed and transported by the plant to reproductive organs. Aerially applied chemicals are absorbed through the foliage. Both applications have the potential to permit the chemical to reach pollen and/or nectar [125].

The application of pesticides has been linked to a decline in resin collection visits, as it results in alterations to the odour (a combination of terpenes) of a resin [8,128]. Stingless bees utilize specific combinations of volatile mono- and sesquiterpenes to identify and differentiate resin sources, and thus, exposure to pesticides may disrupt this process [129].

*Tetragonisca angustula*, *Scaptotrigona postica*, and *Melipona scutellaris* are particularly susceptible to the effects of oral exposition of thiamethoxam, a neonicotinoid pesticide [130]. A study investigating the prevalence of organochlorine pesticides in honey and pollen of *Scaptotrigona mexicana* revealed that 88.44% of honey samples tested positive for at least one organochlorine pesticide. In comparison, only 22.22% of pollen samples showed evidence of contamination [131]. The most prevalent pesticides were heptachlor, γ-HCH, DDT, endrin, and DDE [131].

Few studies have reported the presence of pesticides in other stingless bee products [24,132,133], but there are almost none in matrices such as cerumen or geopropolis. This study’s lack of detection of other pesticides in cerumen samples may be attributed to the prolonged cold storage period (maximum 4 years), which may have rendered the molecules undetectable. However, this does not explain the negative results observed in the fresher samples, which also exhibited no evidence of other pesticide contamination than glyphosate and its AMPA metabolite. Thus, in light of the absence of other pesticides than glyphosate and its AMPA metabolite in cerumen in the present study, it can be postulated that cerumen from Ecuadorian stingless bee species from the genera *Melipona*, *Scaptotrigona*, *Partamona*, *Nanotrigona*, *Paratrigona*, *Trigona*, and *Tetragonisca* from the ten specified Ecuadorian localities do not function as a matrix for the bioaccumulation of the 16 other agrochemicals selected for quantification in Table 2.

It was necessary to incorporate an additional factor to account for the glyphosate risk due to the dilution of detected chemical contaminants resulting from the mixing of cerumen samples in the methodology. However, there are two potential limitations to this approach. Firstly, there is a possibility that a single contaminated sample may have had sufficient chemical concentration to contaminate the potentially negative samples. Secondly, an overestimation of chemical contaminant concentration may have occurred when more than one contaminated sample was present within the same pool.

The susceptibility of different stingless bee species to a chemical contaminant is subject to interspecific variability [134]. The latter was supported by the concentrations of glyphosate, as documented in the literature, which demonstrated lethal effects on three species of stingless bees. In addition to the limited information on LD50 and LC50 of the herbicide in other species of stingless bees and the lack of information for equatorial species, another limitation was noted in the concentrations of glyphosate used in the literature studies.

In a study conducted by Seide et al. (2018) [135], 100% mortality was observed in stingless bee larvae reared in vitro with 3 µL of glyphosate. Given that the acceptable mortality level for stingless bees is 15% [136], the impact of glyphosate on *M. quadrafasciata* larvae appears to be considerable. Further information is required to refine the RQ estimation. This could be achieved by utilizing a range of doses and decreasing concentrations of glyphosate, commencing with the Seide et al. (2018) [135] dose and extending to subsequent dilutions.

In considering the potential impact on humans, it is essential to acknowledge the variability between individuals in terms of their consumption and/or skin contact with cerumen that may be contaminated [137].

To mitigate the potential risks and long-term effects associated with the presence of glyphosate, AMPA, trace metals, and metalloids on stingless bee larvae and adult bees, we propose the implementation of environmentally sustainable agricultural practices among meliponicultors and farmers in surrounding areas where native stingless bees are managed. This approach could facilitate the establishment of mutually beneficial agreements, aligning with the One Health vision.

The reduction in pesticide application, both individually and in cocktails, can be achieved through the establishment of agreements between meliponicultors and farmers for pesticide application, and the implementation of measures to prevent stingless bees from receiving pesticides. These include the relocation of nests or the utilization of quarantine with nests as a preventive measure, which contributes to improving overall management.

Further study of pesticide residues in other stingless bee products is recommended, as well as species-specific studies on the effects of both direct and indirect short- and long-term exposure.

Further studies on lethal doses in each stingless bee species of different pesticides, including glyphosate, are also recommended.

The estimation of consumption and/or skin contact of stingless bee products by humans is also important, and further studies are recommended on the possibility that contaminated matrices such as honey, pollen, or cerumen may be pathways for human health risks.

It is urgent to establish guidelines and safe values that regulate the production and commercialization of stingless bee products in Ecuador and the rest of the countries where Meliponiculture is rapidly entering the market for human consumption and/or skin contact.

## 5. Conclusions

It appears that cerumen may be an effective matrix for monitoring environmental contaminants, including glyphosate (GLY), AMPA, metals, and metalloids. However, it is not the optimal stingless bee product for pesticide monitoring.

Certain levels of glyphosate, cadmium (Cd), chromium (Cr), and lead (Pb) in the cerumen of stingless bees have been identified as exceeding the safe guidelines set out in European and Brazilian legislation for honey bee and stingless bee honey, respectively. Nevertheless, determining their potential hazards remains challenging without Ecuador establishing guidelines specific to this type of matrix.

Given the sampling environment of the stingless bee nests in the northern and southern highlands of the country, it is reasonable to conclude that agriculture and the intensive use of pesticides are the main sources of contamination in products such as cerumen.

The degradation of the environments in which stingless bees naturally live poses an emerging threat to the survival of these species, as well as to food security in relation to the use of their products for human health purposes.

## Figures and Tables

**Table 1:** Sampling sites and the organization of cerumen pools.

Ecuadorian Region	Province	Localities	N°. Pool	N°. Samples Within the Pool	Stingless Bee Genera *
Southern Highlands	Loja (mostly crop environment)	Naranjo, Huertas, Faique, Algarrobillo	1	10	*Scaptotrigona* (58%) *Melipona* (42%)
		Caminuma, Panecillo	2	5	*Scaptotrigona* (81%) *Melipona* (19%)
		Arenal	3	7	*Scaptotrigona* (74%) *Nanotrigona* (14%) *Paratrigona* (12%)
			4	3	*Scaptotrigona* (65%) *Melipona* (35%)
Northern Highlands	Imbabura (crop area and secondary forest)	Intag	5	2	*Partamona* (50%) *Trigona* (50%)
			6	3	*Partamona* (100%)
Low Amazon	Napo (urban environment-Archidona) (primary and secondary forest-Agua Santa)	Archidona	7	2	*Melipona* (50%) *Nanotrigona* (50%)
			8	1	*Melipona* (100%)
		Agua Santa	9	4	*Tetragonisca* (83%) *Nanotrigona* (17%)
			10	1	*Scaptotrigona* (100%)
			11	1	*Melipona* (100%)
			12	1	*Trigona* (100%)
			13	1	*Scaptotrigona* (100%)
			14	1	*Melipona* (100%)

* Images of genera of stingless bees from Ecuador were included in Appendix A.

**Table 2:** Description of pesticides used in Ecuador and selected for analysis.

Name	Type	Group	* CAS Number	Main Crop Use in Ecuador
Acetamiprid	Insecticide	Neonicotinoid	135410-20-7	Vegetable, fruit, and ornamental crops
Carbendazim (benomyl)	Fungicide	Benzimidazole	10605-21-7	Roses, rice, bananas, coffee crops
Chlorantraniliprole	Insecticide	Ryanoids	500008-45-7	Corn crops
Clothianidin	Insecticide	Neonicotinoid	210880-92-5	Tomato, broccoli, roses
Cypermethrin	Insecticide	Pyrethroids	52645-53-1	Corn and broccoli crops
Deltamethrin	Herbicide, Acaricide	Pyrethroids	52918-63-5	Potatoes, grapes, and rose cultivation
Diafenthiuron	Herbicide	Sulfonylurea compound	80060-09-9	Tomatoes, beans, roses
Diazinon	Insecticide	Organophosphorus	333-41-5	Roses, rice, fruits
Flonicamid	Insecticide	Pyridine carboxamides	158062-67-0	Roses, tomatoes
Glyphosate + AMPA (its metabolite)	Herbicide and crop desiccant	Organophosphorus	1071-83-6, 1066-51-9	Weeds, perennial shrubs
Imidacloprid	Insecticide	Neonicotinoid	138261-41-3	Potatoes, corn, fruits, and vegetable crops
Malathion (malaoxon)	Insecticide	Organophosphorus	121-75-5	Rice and corn crops
Methomyl	Insecticide	Carbamates	16752-77-5	Rice and corn crops
Pyrethrin I	Insecticide	Pyrethrins	8003-34-7	Avocado, blueberry, potatoes
Thiacloprid	Insecticide	Neonicotinoid	111988-49-9	Roses crops
Thiamethoxam	Insecticide	Neonicotinoid	153719-23-4	Potatoes, African palm, and cocoa crops
Trichlorfon	Insecticide, Anthelmintic	Organophosphorus	52-68-6	Human and animal drugs

* CAS number: unique identification number, assigned by the Chemical Abstracts Service (CAS).

**Table 3:** The concentration of exposure to glyphosate in the stingless bee *T. angustula*.

Stingless Bee Species	Glyphosate (GLY) Concentration (LD_50_)	Time of Exposure Until 100% of Death	Type of Exposure	Stingless Bee Stage	Reference
*Tetragonisca angustula*	0.015 µg a.i./bee (95% CI: 0.005–0.04) (GLY mixed with honey/water)	48 h	Orally	Adult bees (foragers)	[61]

a.i., active ingredient; CI: confidence interval; µg: micrograms; h: hours.

**Table 4:** Chemical contaminants detected in cerumen pools.

		Highlands						Amazon								Basic Sample Statistics					Permitted Levels		
	Pool	1	2	3	4	5	6	7	8	9	10	11	12	13	14	Av ± SD	Min	Max	No	%	Brazil. Legis. for SB	EU. Guid. for HB	WHO for Humans
ChC																							
Sb			0.04	0.063	0.063	0.038		0.028								0.046 ± 0.016	0.028	0.063	5	36			
As		0.083	0.14	0.14	0.087	0.25	0.27			0.064		0.028				0.133 ± 0.087	0.028	0.27	8	57	0.30	1	
Cd		0.025	0.64	4.4	1.4	0.1	0.048	0.018	0.009	0.1	0.068	0.093	0.034	0.056	0.051	0.503 ± 1.184	0.009	4.4	14	100	0.10		0.008
Cr		0.34	1.7	0.74	0.32	7.1	6	0.11	0.25	3.3	0.29	0.66	0.22	0.15	0.21	1.528 ± 2.304	0.11	7.1	14	100	0.10		0.00013–0.0003.
Sn						0.17										0.170	0.17	0.17	1	7			
Ni		0.13	0.52	0.63	0.3	1.8	1.4	0.093	0.08	0.95	0.13	0.37	0.71	0.48	0.19	0.556 ± 0.518	0.08	1.8	14	100	5		0.5
Pb		0.16	0.26	0.32	0.13	1.5	0.35	0.044	0.031	0.23	0.036	0.082	0.034		0.035	0.247 ± 0.393	0.031	1.5	13	93	0.30	0.10	0.05
Se				0.16												0.160	0.16	0.16	1	7			
GLY		0.02	0.014	0.15		0.2										0.096 ± 0.094	0.014	0.2	4	29	0.05	0.05	0.5
AMPA						0.028										0.028	0.028	0.028	1	7		0.2	

All values are concentrations in mg/kg. Blank spaces represent a numerical value under the Limit of Quantification (LOQ) or absence of limit values. Sb = Antimony (LOQ = 0.02 mg/kg); As = Arsenic (LOQ = 0.02 mg/kg); Cd = Cadmium (LOQ = 0.008 mg/kg); Cr = Chromium (LOQ = 0.02 mg/kg); Sn = Tin (LOQ = 0.1 mg/kg); Ni = Nickel (LOQ = 0.02 mg/kg); Pb = Lead (LOQ = 0.02 mg/kg); Se = Selenium (LOQ = 0.1 mg/kg); AMPA = Aminomethylphosphonic acid (LOQ = 0.01 mg/kg); GLY = Glyphosate (LOQ = 0.01 mg/kg). ChC = Chemical contaminant, Av = average; SD = standard deviation; Min = minimum value; Max = maximum value; No. = number; % = percentage. Permitted levels of contaminants: Brazilian legislation applicable to honey produced by stingless bees (Brazil Legis. for SB) [76,77], European guidelines for honey of *Apis mellifera* (EU. Guid. for HB) [78,79], World Health Organization’s maximum level of daily ingestion of contaminants for humans (WHO for humans) [80].

**Table 5:** Summary of the *p*-value adjusted with the Bonferroni correction for multiple comparison tests between chemical contaminants’ mean ranks and sampling locations.

	Low Amazon— Northern Highlands	Low Amazon— Southern Highlands	Northern—Southern Highlands
Glyphosate			0.1797
Antimony	1	0.2754	0.7831
Arsenic	0.0411 ^a^	0.4544	0.4644
Cadmium	1	0.2237	1
Chromium	0.0378 ^b^	0.4297	0.6425
Nickel	0.0805	1	0.2344
Lead	0.0255 ^c^	0.1603	0.8981

^a,b,c^ significant differences (*p* < 0.05).

**Table 6:** Univariate analysis. Location (independent variable) versus each chemical contaminant detected (dependent variable).

Variable	Estimated Coefficient	Standard Error	Z	*p*-Value	95% Confidence	
					Lower Limit	Upper Limit
Glyphosate	−5.59	3.82	−1.462	0.169	−13.07	1.90
Antimony	−25.79	7.97	−3.36	0.005 ^a^	−40.82	−10.76
Arsenic	−5.83	2.27	−2.57	0.024 ^b^	−10.28	−1.39
Cadmium	−0.44	0.18	−2.42	0.033 ^c^	−0.79	−0.08
Chromium	−0.06	0.11	−0.52	0.616	−0.28	0.16
Nickel	−0.23	0.50	−0.46	0.658	−1.22	0.76
Lead	−0.68	0.66	−1.03	0.325	−1.97	0.62

^a,b,c^ significant differences (*p* < 0.05).

**Figure A1:**
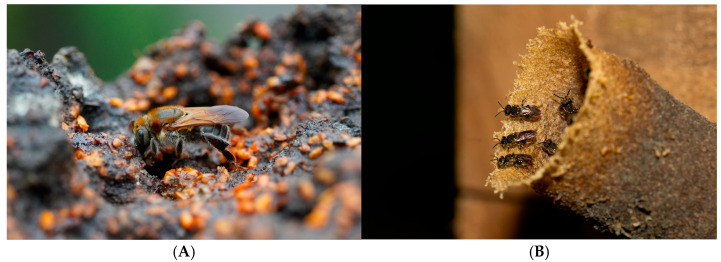

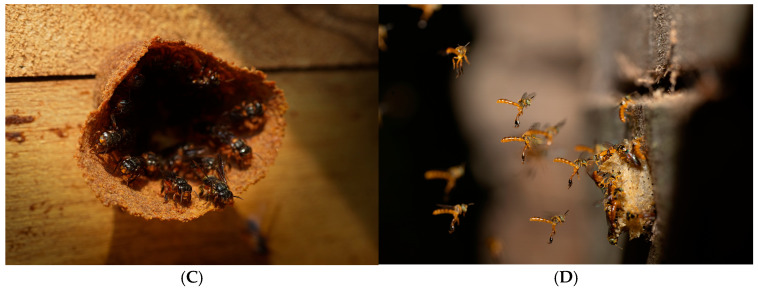
(**A**) *Melipona grandis*; (**B**) *Nanotrigona* sp.; (**C**) *Scaptotrigona* sp.; (**D**) *Tetragonisca angustula*. Fundación Yachana. Napo, Ecuador. Author: Alfonso Jiménez.

## Data Availability

The original contributions presented in this study are included in the article. Further inquiries can be directed to the corresponding author.

## Appendix A

The following images of stingless bees from Ecuador were included in the cerumen study.

## Appendix B

LC-MSMS detection parameters, and detailed validation data for multi-residue analysis

### Appendix B.1. Chromatographic Parameters

Eluent A: ultrapure water + 0.1% acetic acid + 5 mM ammonium acetate

Eluent B: LC-MS grade methanol + 0.1% acetic acid + 5 mM ammonium acetate

Flow rate: 0.7 mL/min

Oven temperature: 60 °C

Injection volume: 10 µL

Gradient:

**Table A1:** Time and eluent conditions for the LC-MSMS.

Time (min)	% Eluent A	% Eluent B
0	80	20
0.1	80	20
1	50	50
9	20	80
12	0	100
13	0	100
13.5	80	20
15	80	20

### Appendix B.2. Detection Parameters

**Table A2:** Summary of parameters obtained from the mass spectrometer.

Group ID	Compound ID	Q1 Mass (Da)	Q3 Mass (Da)	Dwell Time (ms)	EP (V)	CE (V)	CXP (V)
**Positive Ion Electrospray Ionization**							
Acetamiprid	acetamiprid_1	223.2	126	20	10	27	15
Acetamiprid	acetamiprid_2	223.2	90.1	20	10	45	15
Benomyl	benomyl_1	291	192	20	10	17	10
Benomyl	benomyl_2	291	160	20	10	39	8
Carbendazim	carbendazim_1	192.1	160.1	20	10	25	15
Carbendazim	carbendazim_2	192.1	132.1	20	10	41	15
Methomyl	methomyl_1	163	88	20	10	13	14
Methomyl	methomyl_2	163	106.1	20	10	15	8
Flonicamid	flonicamid_1	230.1	203.1	20	10	25	12
Flonicamid	flonicamid_2	230.1	174.05	20	10	27	8
Thiacloprid	thiacloprid_1	253	126	20	10	29	15
Thiacloprid	thiacloprid_2	253	186	20	10	19	15
Thiamethoxam	thiamethoxam_1	292	211	20	10	17	10
Thiamethoxam	thiamethoxam_2	292	181	20	10	33	8
Malaoxon	malaoxon_1	315	127.1	20	10	17	15
Malaoxon	malaoxon_2	315	99.2	20	10	31	15
Malathion	malathion_1	331	127	20	10	17	29
Malathion	malathion_2	331	284.9	20	10	11	16
Chlorantraniliprole	chlorantraniliprole_1	484	453	20	10	23	12
Chlorantraniliprole	chlorantraniliprole_2	484	286	20	10	19	18
Trichlorfon	trichlorfon_1	257	108.9	20	10	27	18
Trichlorfon	trichlorfon_2	257	220.8	20	10	17	14
Pyrethrin I	pyrethrin I_1	329.179	161.2	20	10	15	14
Pyrethrin I	pyrethrin I_2	329.179	143.2	20	10	25	10
Pyrethrin I	pyrethrin I_3	329.179	133.1	20	10	25	14
Imidacloprid	imidacloprid_1	256.007	209.1	20	10	23	12
Imidacloprid	imidacloprid_2	256.007	175.2	20	10	29	10
Diafenthiuron	diafenthiuron_1	385.238	329.148	20	10	26	7
Diafenthiuron	diafenthiuron_2	385.238	287.05	20	10	36	6
Diafenthiuron	diafenthiuron_3	385.238	262.045	20	10	64	8
Diafenthiuron	diafenthiuron_6	385.2	278.2	20	10	45	8
**Negative Ion Electrospray Ionization**							
Clothianidin	clothianidin_1	247.873	57.9	20	−10	−16	−9
Clothianidin	clothianidin_2	249.966	58.1	20	−10	−16	−7

## Appendix C

GC-MSMS detection parameters, and detailed validation data for multi-residue analysis

### Appendix C.1. Chromatographic Parameters

**Table A3:** Main conditions used in GC-MSMS.

Carrier Gas	Helium
Constant flow	1 mL/min
Oven temperature	60 °C
Injection mode	Solvent vent
Injection volume	1 µL

**Table A4:** Injector program.

Temperature (°C)	Ramp (°C/min)	Hold (min)	Total (min)
45	-	0.02	0.02
325	600	5	18.497

**Table A5:** Oven program.

Temperature (°C)	Ramp (°C/min)	Hold (min)	Total (min)
45	-	1.0	1.0
170	45.5	0.0	3.7473
310	10	0.75	18.497
310 (backflush)	2.0 (backflush)	2.0 (backflush)	20.497 (backflush)

### Appendix C.2. Detection Parameters

**Table A6:** Parametres of the tandem mass spectrometry experiment.

Molecule	N° Transition	Precursor Ion (*m*/*z*)	Product Ion (*m*/*z*)	CE (V)
Diazinon	2	199.1	135.1	10
Diazinon	1	137.1	84	10
Cypermethrine	2	164.9	127	5
Cypermethrine	1	163	127	5
Deltamethrine	1	252.9	174	5
Deltamethrine	2	250.7	172	5

### Appendix C.3. Validation Data

Validation 5 recoveries at LOQ + 5 recoveries at 10×LOQ.

**Table A7:** Main statistics during the validation data process.

Molecule	LOQ (µg/kg)	Mean Recovery (%)	RSD (%)
Acetamiprid	10	75	8
Benomyl	10	-	-
Carbendazim	10	64	14
Chlorantraniliprole	10	67	7
Clothianidin	10	65	5
Cypermethrin	20	123	10
Deltamethrin	10	73	14
Diafenthiuron	50	-	-
Diazinon	10	65	17
Flonicamid	20	63	3
Imidacloprid	50	59	12
Malaoxon	20	76	7
Malathion	10	70	4
Methomyl	10	64	5
Pyrethrin I	50	63	13
Thiacloprid	20	69	5
Thiamethoxam	10	56	8
Trichlorfon	10	88	16

## Appendix D

LC-MSMS detection parameters and detailed validation data for glyphosate and AMPA analysis.

### Appendix D.1. Chromatographic Parameters

**Table A8:** Eluent parameters in LC-MSMS.

Eluent A	0.1 mol/L ammonium bicarbonate in ultrapure water/ammonium hydroxide (100/0.1; *v*/*v*)
Eluent B	Ultrapure water/formic acid (100/0.02; *v*/*v*)
Flow rate	0.7 mL/min
Oven temperature	40 °C
Injection volume	40 µL

### Appendix D.2. Gradient

**Table A9:** Time and eluent conditions for the LC-MSMS.

Time (min)	% Eluent A	% Eluent B
0	10	90
0.5	10	90
0.6	20	80
2	20	80
7	50	50
10	50	50
10.5	100	0
11.5	100	0
11.6	10	90
14	10	90

### Appendix D.3. Detection Parameters

**Table A10:** Summary of parameters obtained from the mass spectrometer.

Q1	Q3	Dwell Time	Pesticide	DP	CE	CXP
110	63	80	AMPA_1	−20	−25	−8.5
110	79	80	AMPA_2	−20	−35	−10
168	63	40	glyphosate_1	−25	−28	−6.7
168	81	40	glyphosate_2	−25	−21	−8
168	150	40	glyphosate_3	−25	−14	−7

### Appendix D.4. Validation Data

Validation 5 recoveries at LOQ + 5 recoveries at 10×LOQ

**Table A11:** Main statistics during the validation data process.

Molecule	LOQ (µg/kg)	Mean Recovery (%)	RSD (%)
AMPA	10	105	8
Glyphosate	10	106	8

## Appendix E

IC-MS detection parameters and detailed validation data for metals and metalloids trace analysis.

### Appendix E.1. General Detection Parameters

**Table A12:** Gas injection parametres.

Nebulising gas	Argon at 1.08 L/min
Auxiliary gas	Argon at 0.90 L/min
Plasma gas	Argon at 15 L/min
Collision gas	Helium

**Table A13:** Isotope daltons used for each metals and metalloid.

												ISTD		
	Metalloids			Metals										
	Sb	As	Sc	Cd	Cr	Cu	Sn	Hg	Ni	Pb	Se	Sc	Ir	Rh
Isotope (Da)	121	75	45	111	52	63	118	201 + 202	60	206,207,208	78	45	193	103

### Appendix E.2. Validation Data

5 recoveries at LOQ + 5 recoveries at 10×LOQ

**Table A14:** Main statistics during the validation data process.

	LOQ (µg/kg)	Mean Recovery (%)	RSD (%)
Metals			
Cd	8	102	5
Cr	20	101	3
Cu	125	113	8
Hg	20	102	3
Ni	20	104	5
Pb	20	101	5
Se	100	107	9
Sn	100	103	4
Metalloids			
Sb	20	95	12
As	20	105	7
